# The relation between age and airway epithelial barrier function

**DOI:** 10.1186/s12931-022-01961-7

**Published:** 2022-03-03

**Authors:** M. de Vries, K. O. Nwozor, K. Muizer, M. Wisman, W. Timens, M. van den Berge, A. Faiz, T.-L. Hackett, I. H. Heijink, C. A. Brandsma

**Affiliations:** 1 University Medical Center Groningen, University of Groningen, Department of Epidemiology, Hanzeplein 1, 9713 Groningen, The Netherlands; 2 University Medical Center Groningen, University of Groningen, Groningen Research Institute for Asthma and COPD, Groningen, The Netherlands; 3 University Medical Center Groningen, University of Groningen, Department of Pathology and Medical Biology, Groningen, The Netherlands; 4 University Medical Center Groningen, University of Groningen, Department of Pulmonary Diseases, Groningen, The Netherlands; 5 Department of Anesthesiology, Pharmacology & Therapeutics, Centre for Heart Lung Innovation, The University of British Columbia, Vancouver, Canada

**Keywords:** Ageing, Epithelial barrier function, *EPCAM*, *CDH1*, Airway epithelial cells, Chronic airway diseases

## Abstract

**Background:**

The prevalence of age-associated diseases, such as chronic obstructive pulmonary disease (COPD), is increasing as the average life expectancy increases around the world. We previously identified a gene signature for ageing in the human lung which included genes involved in apical and tight junction assembly, suggesting a role for airway epithelial barrier dysfunction with ageing.

**Aim:**

To investigate the association between genes involved in epithelial barrier function and age both in silico and in vitro in the airway epithelium.

**Methods:**

We curated a gene signature of 274 genes for epithelial barrier function and tested the association with age in two independent cohorts of bronchial brushings from healthy individuals with no respiratory disease, using linear regression analysis (FDR < 0.05). Protein–protein interactions were identified using STRING©. The barrier function of primary bronchial epithelial cells at air–liquid interface and CRISPR–Cas9-induced knock-down of target genes in human bronchial 16HBE14o-cells was assessed using Trans epithelial resistance (TER) measurement and Electric cell-surface impedance sensing (ECIS) respectively.

**Results:**

In bronchial brushings, we found 55 genes involved in barrier function to be significantly associated with age (FDR < 0.05). *EPCAM* was most significantly associated with increasing age and *TRPV4* with decreasing age. Protein interaction analysis identified *CDH1*, that was negatively associated with higher age, as potential key regulator of age-related epithelial barrier function changes. In vitro, barrier function was lower in bronchial epithelial cells from subjects > 45 years of age and significantly reduced in *CDH1*-deficient 16HBE14o-cells.

**Conclusion:**

The significant association between genes involved in epithelial barrier function and age, supported by functional studies in vitro*,* suggest a role for epithelial barrier dysfunction in age-related airway disease.

**Supplementary Information:**

The online version contains supplementary material available at 10.1186/s12931-022-01961-7.

## Background

As human life expectancy increases, the age of the general population rises. As a consequence, the prevalence of age-associated diseases, such as chronic obstructive pulmonary disease (COPD) is also increasing, resulting in a major health and societal burden around the world [1, 2]. To intervene with these age-associated diseases, it is important to understand the underlying mechanisms and consequences of ageing.

Ageing is described as a time-dependent accumulation of cellular damage which results in a gradual decline in overall function, leading to increased risk of diseases and death [3, 4]. One of the organs that is highly impacted by ageing is the lung [5]*.* An important feature of the ageing lung is the gradual decline in lung function [4], which is measured by a gradual decrease in forced expiratory volume in 1 s (FEV_1_) and Forced Vital Capacity [6, 7]. This decline in lung function is due to structural and physiological changes in the lung, including the destruction of alveolar septa, loss of elastic recoil and airspace enlargement; a phenomenon known as senile emphysema [4]. Other features of lung ageing are ‘immunosenescence’ and ‘inflam-aging’, conditions characterized by abnormal immune responses following an infection or injury, which may contribute to higher susceptibility towards infections and increased lung damage with ageing [2, 4].

We recently showed that gene expression in human lung tissue is strongly associated with chronological age [8]. Two of the highest enriched biological processes amongst the genes that decreased with increasing age were those associated with the assembly of tight and apical junctional complexes. Since the genes involved in these processes are important regulators of airway epithelial barrier formation, we hypothesized that airway epithelial barrier function is reduced with ageing. The airway epithelium is the first line of defence against the inhaled environment. It forms a physical barrier that protects the submucosal tissue from pathogens and other harmful substances in the inhaled air [9]. Epithelial barrier function is maintained by the formation of tight and adherens junctions between adjacent cells, in which the transmembrane protein E-cadherin plays a crucial role [10].

The aim of this study was to identify the key genes involved in age-related changes in epithelial barrier function and to gain insight in the functional consequences of age on the airway epithelial barrier. Therefore, we first investigated, in silico*,* the association between age and the expression of genes involved in epithelial barrier function. Secondly, we studied, in vitro*,* the effect of age on airway epithelial barrier function in airway epithelial cells.

## Methods

### In silico study

#### Study population

Bronchial airway brushings were obtained during bronchoscopy from two independent cohorts. The first cohort was a combination of subjects with normal pulmonary function from the study to obtain normal values of inflammatory variable from healthy subjects (NORM) and the Top Institute Pharma (TIP) project (N = 147), both conducted at the University Medical Center Groningen [11]. (The NORM and TIP cohorts are subsequently referred to as Groningen cohort). The second cohort was a publicly available dataset deposit in the Gene Expression Omnibus (GSE37147), including ex- and current smoking patients with COPD and non-COPD controls [12]. For this study, we selected only the non-COPD controls (N = 151) as defined by Steiling et al. [12]. In both datasets (Groningen and GSE37147), RNA was isolated from bronchial airway brushes and gene expression levels were measured with the Affymetrix Human Gene 10 ST Array.

#### Gene expression signature and age-association analysis

To study the age-related association of genes involved in epithelial barrier function in bronchial brushes, we first selected relevant genes for epithelial barrier function. We used the Molecular Signature Database v7.1 and selected the genes included in the Gene Ontology datasets “Apical junction complex”, “Tight junction” and “Adherens junction” [13–15]. Genes present in more than one dataset were only included once.

Linear regression analysis was performed using R software 3.5.0 in both datasets separately. The analyses were adjusted for sex and smoking status. To adjust for unwanted technical variation, we included principal components explaining more than 1% of the variation in each dataset. A random-effect meta-analysis was performed to determine which genes were associated with age in both datasets. To correct for multiple testing, Benjamini–Hochberg false discovery rate (FDR < 0.05) was applied.

#### Identification of key regulatory genes of epithelial barrier function

To identify key regulatory genes of epithelial barrier function, we assessed the protein–protein interactions using STRING© Consortium 2018V.10.5 for the genes significantly associated with age in the meta-analysis [16].

### In vitro study

#### Cell cultures

Primary bronchial epithelial cells (PBECs) obtained from bronchial brushes from healthy subjects were cultured at air–liquid interface (ALI) (n = 14, age range 19–70 years).

The human bronchial epithelial cell line (16HBE14o-) was used to generate heterozygous (*CDH1*^+/−^) and homozygous (*CDH1*^−/−^) CRISPR–Cas9 E-cadherin knockout cell lines (details in Additional file 1).

#### In vitro experiments

TER was measured in fully differentiated ALI cultures, cultured 21–25 days, described in a previous study by *de Vries *et al. [17] and in Additional file 1. RNA was isolated from primary epithelial cells from eight donors following 21 days in ALI culture. cDNA was synthesized and expression of *CDH1* was assessed using Taqman assay and normalized against the average expression of the housekeeping genes *B2M* and *PPIA*.

The electrical resistance of wild-type (WT) 16HBE14o-, *CDH1*^+/−^ and *CDH1*^−/−^ cells was measured real-time for 48 h using electric cell-substrate impedance sensing (ECIS, Applied Biophysiscs, Troy, NY, USA), at a frequency of 400 Hz as previously described [18].

#### Statistical analysis of in vitro experiments

Differences in TER between ALI cultures from subjects below 45 years of age and above 45 years of age were assessed using the Mann–Whitney U test. Associations between age and experimental read-outs were tested using spearman correlation. Differences between groups in epithelial resistance over time were assessed by 2-way ANOVA. All statistics were performed using GraphPad Prism v6 and a p-value of 0.05 was considered significant.

## Results

### Subject characteristics for bronchial brushings cohorts

An overview of the clinical characteristics of the subjects included in both bronchial brushing cohorts is presented in Table 1. The Groningen cohort consisted of 147 current, ex- and never-smoking respiratory healthy controls with an age range of 18–73 years. The GSE37147 cohort consisted of 151 current and ex-smoking respiratory healthy controls with an age range of 48–77 years.

**Table 1:** Subject characteristics

	Groningen	GSE37147
Number	147	151
Age, years (range)	37 (18–73)	64 (48–77)
Male/female, N	91/56	83/68
Smoking, N		
Never	47	0
Ex	6	82
Current	94	69
Pack years, N	17 (15)^a^	47 (19)^b^
FEV_1_, %predicted	109 (11.7)	93 (13)
FEV_1_/FVC, %	82.6 (6)	75 (6)

Data are presented as mean with standard deviation, unless otherwise stated

*FEV*_*1*_ forced expiratory volume in 1 s, *FVC* forced vital capacity

^a^Packyears was missing for 12 subjects excluding never smokers

^b^Packyears were missing for 11 subjects

### Gene signature for epithelial barrier function

**Fig. 1:**
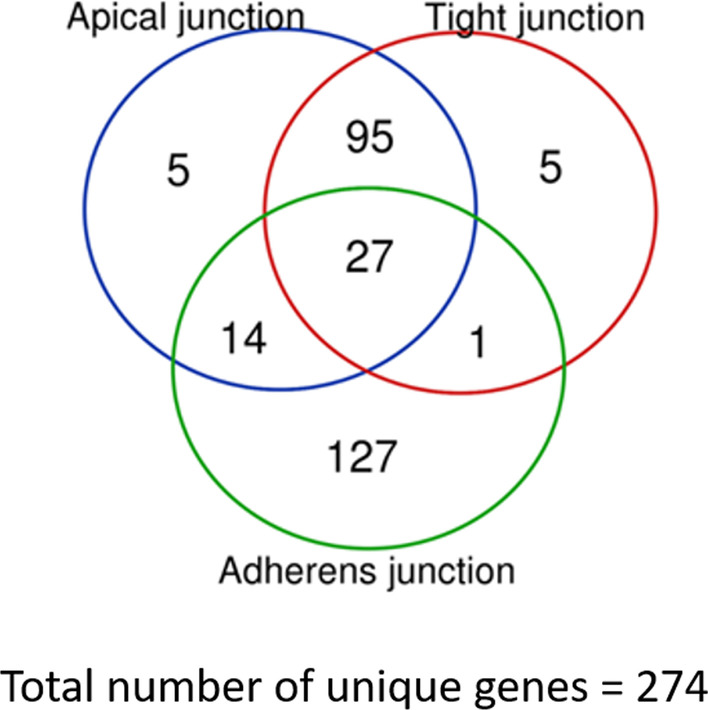
Venn diagram summarizing the age-related gene signature of airway epithelial barrier function. In total, 274 unique genes were present in the three biological processes. 141 Genes in apical junction complex, 128 in tight junction assembly and 168 present in adherens junction assembly

First, we curated a signature of epithelial barrier function genes using the Gene Ontology datasets “Apical junction complex”, “Tight junction” and “Adherens junction”. The apical junction complex dataset consisted of 141 genes, the tight junction dataset consisted of 128 genes and the adherens junction dataset was composed of 169 genes. After combining the genesets, our gene signature for epithelial barrier function included 274 unique genes (Additional file 2: Table S1). As shown in the Venn diagram in Fig. 1, 27 genes were present in all three datasets, 110 genes in two datasets and 137 genes only in one dataset.

### Age-related gene expression signature of epithelial barrier function in bronchial brushings

**Fig. 2:**
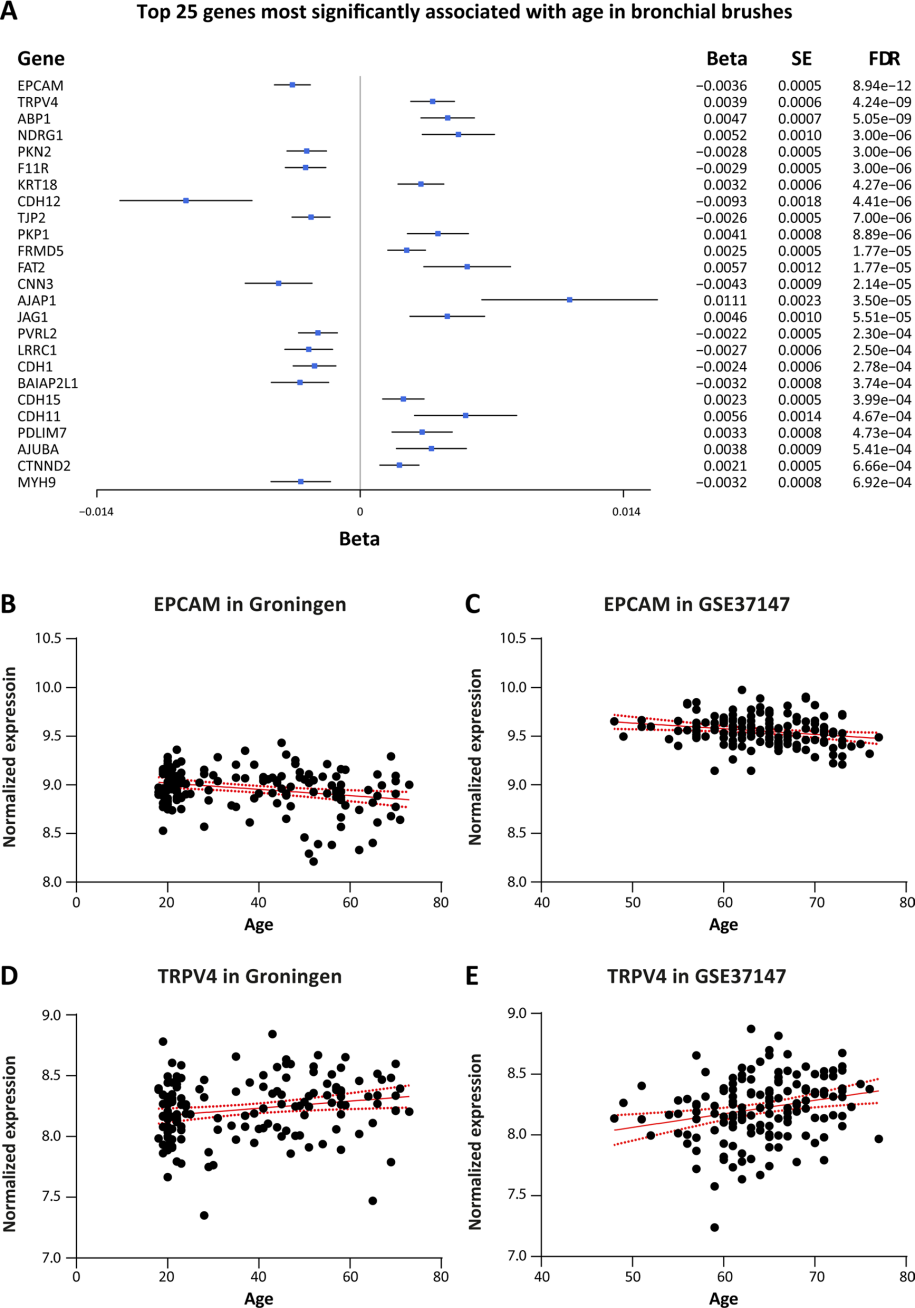
Overview of the top-results of the age-related gene expression signature of epithelial barrier function in bronchial epithelial cells. **A** Forest plot showing the effect estimates (beta) and standard errors (SE) of the top 25 genes involved in the epithelial barrier function of the meta-analysis on the Groningen and GSE37147 cohorts. **B**, **C** Expression of *EPCAM* and age in Groningen and GSE37147. **D**, **E** Expression of *TRPV4* and age in Groningen and GSE37147

Of the 274 genes included in our epithelial barrier gene signature, 271 were present in the gene expression data of both bronchial brush cohorts (Groningen and GSE37147). The meta-analysis showed that 55 of these genes were significantly associated with age (FDR adjusted p-value < 0.05). Of these genes, 25 had lower expression with increasing age, while 30 had higher expression with increasing age. The most significantly decreased gene with increasing age was *EPCAM *(epithelial cell adhesion molecule) (FDR = 8.94e−12). The most significantly increased gene expressed with increasing age was *TRPV4* (transient receptor potential vanilloid 4, FDR = 4.24e−09). An overview of the top 25 epithelial barrier genes that are most significantly associated with age is presented in the forest plot in Fig. 2A. The association between *EPCAM* and *TRPV4* and age in both cohorts is depicted in Fig. 2B–E. In addition, we assessed the association between *EPCAM* and *TRPV4* and age in primary bronchial epithelial cells of 8 healthy donors cultured at air–liquid interface. Although we could not find a significant association with age for both genes, likely due to low sample size, the results for *TRPV4* are in line with our initial gene expression findings (Additional file 4: Fig. S1). A complete overview of the meta-analysis for all 274 genes can be found in Additional file 3: Table S2.

### Protein interaction analysis identifies *CDH1* as potential key regulatory gene

**Fig. 3:**
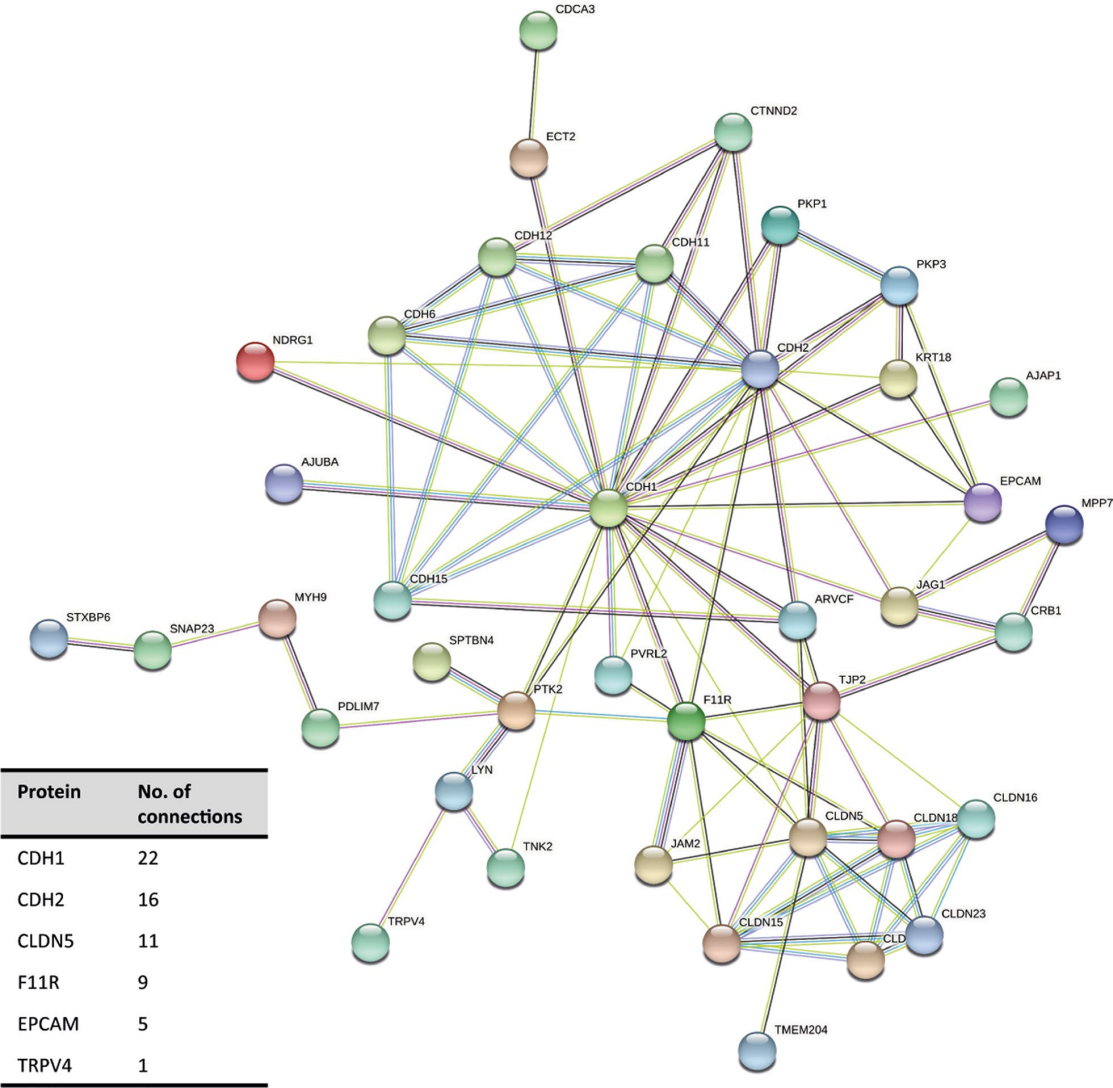
Schematic overview of the protein–protein interactions of the epithelial barrier genes associated with age in bronchial epithelial cells. The 55 genes significantly associated with age were included in the String analysis. Genes with more than one interaction or present in the top 25 most significantly associated with age are shown in the figure. Blue line indicates known interaction from curated databases, purple line indicates known interaction experimentally determined, green line indicates predicted interaction based on gene neighbourhood and black line indicates interaction based on co-expression

STRING© was used to identify potential key regulatory genes of epithelial barrier function with age. The STRING© network, based on the 55 age-related epithelial barrier genes, was significantly enriched for protein–protein interactions (p-value < 1.0 × 10^–16^). *CDH1,* encoding the adherens junction molecule E-cadherin that is highly expressed in airway epithelial cells [19], was identified as a potential key regulatory gene with the highest number of protein–protein interactions in the network (Fig. 3). Off note, EPCAM and TRPV4 were positioned at the periphery of the interaction network with 5 and 1 interactions respectively, thus we focused on CDH1 as a key regulator of epithelial barrier function regulation with age. Therefore, for the subsequent in vitro studies, we focused on the role of E-cadherin.

### Association between age and epithelial barrier function in vitro

**Fig. 4:**
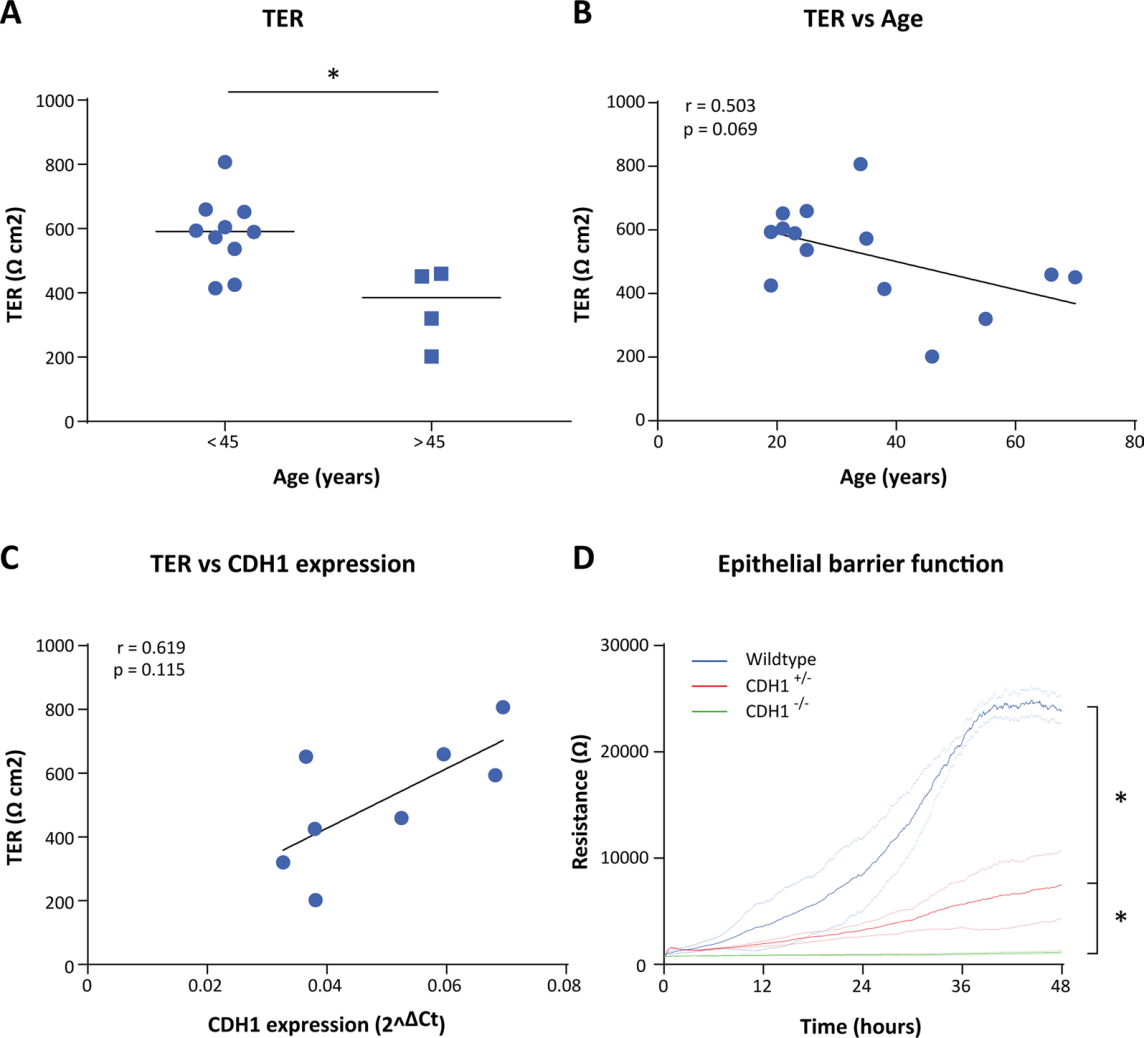
Association between age and epithelial barrier function in vitro. **A** Transepithelial resistance (TER) of fully differentiated air–liquid interface (ALI) cultures of healthy subjects below 45 years of age and above 45 years of age. Primary bronchial epithelial cells (PBECs) were isolated from healthy subjects of different age and cultured at ALI for 21 days and differences between the age group were tested with the Mann–Whitney U test, * = p < 0.05. **B** Spearman correlation between TER of the ALI cultures and age. **C** Spearman correlation between TER of ALI cultures and expression of *CDH1*, presented as the log2 fold of the delta C_t_ value. **D** Real-time measurement of the epithelial resistance of control (blue line), CDH1^+/−^ (red) and CDH1^−/−^ (green) CRISPR–Cas9 induced knockout 16HBE14o-cells for 48 h. The mean of n = 3 for each group is shown, and the faint dotted lines represent the SEM. Differences between the groups were tested with 2way-ANNOVA, * = p < 0.05

Next, we assessed whether age was associated with airway epithelial barrier function. In ALI-differentiated PBECs from healthy donors, we observed significantly lower TER in subjects above 45 years of age compared to subjects below 45 years of age (Fig. 4A). Although not significant, there was a negative association between TER and age (Fig. 4B) and no significant association between TER and *CDH1* (Fig. 4C). To further assess the functional role of *CDH1* in bronchial epithelial barrier formation, we downregulated *CDH1* using CRISPR–Cas9 in human bronchial 16HBE14o-cells. We successfully generated a full knockout (*CDH1*^−/−^) and a heterozygous knock out with one functional allele remaining (*CDH1*^+/−^). The western blot confirming the generation of the CDH1 knock out cell lines is shown in Additional file 5: Fig. S2. Subsequently, we measured the electrical resistance in these cell lines together with a wild type control. As shown in Fig. 4D, the wild-type 16HBE14o-cells formed strong cell–cell contacts within 48 h, as demonstrated by the strong increase in low-frequency resistance values. In contrast, in the presence of one functional allele of *CDH1* (*CDH1*^+/−^), the resistance built up slower with a significantly (p = 0.0018) lower maximal resistance achieved, while in the absence of *CDH1* no low-frequency resistance was build up at all (p = 0.0002). The decrease in high-frequency capacitance in all three cell lines indicated that cells were all able to form a confluent layer (Additional file 6: Fig. S3) and that the impairments in the *CDH1* deficient cells are caused by defective formation of epithelial junction.

## Discussion

In this study, we identified 55 age-related epithelial barrier function genes in the airway epithelium. Of these genes, *CDH1*, which had lower expression with increasing age in our meta-analysis, was identified as potential key regulator of epithelial barrier function. Moreover, our in vitro findings suggest a role for ageing in the reduction of airway epithelial barrier function and show that reduced *CDH1* expression can lead to defects in the formation of an epithelial barrier.

The changes that have been described upon ageing in the lung parenchyma include loss of elasticity, enlargement of airspaces and abnormalities in the immune system [2, 5]. The structural changes contribute to the lung function decline observed with increasing age [4, 20], whereas the immunological changes may result in increased damage to lung tissue. How ageing impacts the airway epithelium has not been widely reported. Angelidis et al. have shown that ciliary beat frequency in the airway epithelium decreases with age in mice [20]. Our data add to these findings by showing differences in airway epithelial barrier genes in human airway epithelial cells upon ageing. Our data suggest that these changes may lead to decreased airway epithelial barrier function, which may result in increased fragility of the epithelial barrier with ageing, potentially leading to increased susceptibility to inhaled insults of the airway epithelium with e.g. smoke exposure. Together with an altered immune response, reduced cough strength and a reduction in mucociliary clearance capacity with ageing [4, 5], loss of physical barrier function may also contribute to the higher susceptibility towards bacterial and viral infections, facilitating their access to submucosal layers [21].

In our analysis, *TRPV4* showed the highest significant association with ageing. TRPV4 is one of the Transient Receptor Potential (TRP) superfamily members, which is expressed in the epithelium of a broad variety of tissues, including the tracheal, bronchial and alveolar epithelium of lungs [22]. TRPV4 has been shown to regulate the balance of intracellular calcium concentration [23], however, not much is known about TPRV4 in relation to airway epithelial barrier function. We speculate that there may be a link between the involvement of TRPV4 in calcium regulation and barrier function, as it has been reported that intracellular increases and extracellular depletion of calcium ion impairs cell–cell contact [18]. Further studies are needed to find a definitive link between this protein and barrier function.

Another interesting finding of our meta-analysis was the highly significant negative association of *EPCAM* with increased age. *EPCAM* encodes an evolutionally conserved type 1 transmembrane glycoprotein expressed on epithelial cells of higher eukaryotes, with an extracellular, short intramembranous and a single intracellular domain [24]. In the lungs, the epithelial cells of the trachea, bronchi, bronchioles and alveoli are all EpCAM positive [25]. The primary function of EpCAM is the regulation of cell–cell interaction, and it interacts with several key cell adhesion molecules, including claudin-1 and -7 [26], which were also found to be lower expressed with ageing (Additional file 3: Table S2), further stressing its importance in cell junction [27]. It was previously thought that EpCAM and E-Cadherin have opposing functions because EpCAM is highly expressed in different carcinomas and its knockdown represses cancer cell proliferation, whereas lower expression of E-cadherin in tumour cells has been found to be associated with epithelial–mesenchymal transition, promoting metastasis [27, 28]. This line of thought has been challenged by other studies which have shown the involvement of EpCAM in tight junction formation in both mouse and human intestinal epithelium [28]. Mutations in the *EPCAM* gene have been shown to cause intestinal barrier and ion transport dysfunction, and deletion of E-cadherin from mouse intestine lead to a compromised intestinal barrier function [28]. It has also been shown that ZEB1, a transcription factor associated with epithelial–mesenchymal transition in cancer progression, is not only a direct transcription repressor of E-cadherin in human breast cancer cell line [29] but also of EpCAM in both human pancreatic and breast cancer cell lines [30]. Together, these data suggest that EpCAM and E-cadherin have a synergistic physiological role.

Similar to *EPCAM*, E-cadherin or *CDH1* expression was lower with higher age in epithelial brushes and we identified *CDH1* as a potential key regulatory gene. As described above, E-cadherin plays a crucial role in maintaining intercellular contacts and ensuring apical-basolateral dichotomy [10]. We have previously shown that E-cadherin is downregulated in the airway epithelium in COPD and is thought to contribute to the epithelial barrier dysfunction observed in COPD [9, 31]. Epithelial barrier dysfunction has also been proposed to contribute to increased susceptibility towards bacterial and viral infection of the lungs [32], and propagate inflammatory responses as well as airway wall remodelling [31]. With the available information on the involvement of EpCAM in epithelial cell–cell adhesion and the already established dependency of tight junction formation on E-cadherin in vitro [10, 31], we can propose that the decline of *EPCAM* and *CDH1* with age may contribute to airway epithelial barrier destabilisation and loss of barrier integrity. However, the order of events in which epithelial cells lose barrier function upon ageing needs to be further established.

Our in vitro data showed that the barrier formation of fully differentiated bronchial epithelial cells was lower in older donors compared to younger donors, suggesting that the ability of the bronchial epithelium to form a tight barrier decreases with age. This could be related to the lower *CDH1* expression. We observed a strong defect in barrier formation in both the partial and complete *CDH1* knockout bronchial epithelial cells. This finding builds further on a previous in vitro study of our group, where we showed that siRNA repression of *CDH1* in the human bronchial epithelial cells results in lower barrier function [33] and confirms that E-cadherin is critical for the formation of epithelial junctions. Moreover, we reported in an epithelial specific E-cadherin knock-out mouse model that loss of *CDH1* in the lung epithelium leads to airway epithelial denudation, reduced expression of tight junction protein zonula occludens (ZO)-1, loss of ciliated cells, increased mucus production and pro-inflammatory epithelial phenotype [34]. These findings again point towards the importance of *CHD1* in the maintenance of airway epithelial integrity.

A possible limitation of this study is low sample size in our in vitro studies. This reduced our statistical power, especially in the association between TER and age and TER and *CDH1*. Nevertheless, the outcomes of our in vitro studies clearly support our in-silico analyses and our hypothesis regarding the impact of age on the epithelial barrier function. For the future, larger studies are warranted to further unravel the mechanisms underlying the age-related changes in the epithelial barrier. In addition, also data on protein expression in ALI differentiated epithelial cells would be of interest.

In conclusion, our study shows an age-related association of epithelial barrier gene expression pattern together with a reduced ability of the bronchial epithelium to form a tight barrier in vitro at older age. This reduced epithelial barrier capacity observed in vitro with ageing may contribute to increased susceptibility for chronic airway diseases, like COPD.

## Supplementary Information

**Additional file 1.** Online data supplement (methods).

**Additional file 2: Table S1.** Gene list used for selection of the genes.

**Additional file 3: Table S2.** Summary statistics of the meta-analysis of the age-related gene expression signature of epithelial barrier function in bronchial brushings.

**Additional file 4: Figure S1.** Expression of EPCAM and TRPV4 in vitro in air-liquid interface cultures.

**Additional file 5: Figure S2.** A representative western blot showing E-cadherin expression in human bronchial epithelial cell line 16HBE14o.

**Additional file 6: Figure S3.** Representative figure for real-time measurement of the epithelial capacitance of wild type (blue), CDH1^+/−^ (red) and CDH1^−/−^ (green) CRISPR–Cas9 induced knockout 16HBE14o- cell lines for 48 h.

## Data Availability

The summary statistics of the performed analyses are included in the additional files of the published article. The datasets used during the current study are available from the corresponding authors on reasonable request.
