# Supplementary material for: The relation between age and airway epithelial barrier function
Source: Respir Res. 2022 Mar 3;23:43. doi: 10.1186/s12931-022-01961-7 (PMC8892715; doi:10.1186/s12931-022-01961-7)
Supplement: Supplementary file 1 — Additional file 1. Online data supplement (methods). [file 12931_2022_1961_MOESM1_ESM.docx]

**THE RELATION BETWEEN AGE AND AIRWAY EPITHELIAL BARRIER FUNCTION**

Vries de M.^a,b*^, Nwozor K.O.^b,c*^, Muizer K.^b,c^, Wisman M. ^b,c^, Timens W.^b,c^, Berge van den M.^b,d^, Faiz A.^b,c,d^, Hackett T-L.^e^, Heijink .I.H. ^b,c,d¶^, Brandsma C.A^b,c¶^

* Co-first authors

^¶^ Co-last authors

**Online data supplement**

**Study population: Groningen and GSE37147 cohorts**

The NORM cohort is a combination of the study to obtain normal values of inflammatory variables from healthy subjects (NORM) and the Top Institute Pharma (TIP) study, consisting of never, ex- and current smoking subjects with normal lung function and patients with COPD (NORM, n=167)[1]. Both studies were approved by the local medical ethics committee of the University Medical Centre Groningen and all participants signed informed consent.

The GSE37147 cohort is a publicly available dataset deposited in the Gene Expression Omnibus (GSE 37147), including a total of 267 ex- and current smoking patients with COPD and non-COPD controls, first described by Steiling et al[2].

We excluded the COPD patients from both datasets for our study. That gave us NORM n=147 and Boston n=151.

***IN VITRO STUDY***

**Culture of human bronchial epithelial cells**

Primary bronchial epithelial cells (PBECs) in passage 2 were seeded on collagen I coated transwells (6,5 mm with 0,4 µM Pore Polyester Membrane Insert, Corning, NY, USA) at a density of 0,7x10^5 cells per transwell in serum-free hormonally-supplemented BEGM. At the same time, 0,5 ml serum-free hormonally-supplemented BEGM was added to the basal compartment. After 24 hours, the apical medium was removed and the basal medium was replaced with 300 µl 1:1 Dulbecco’s modified Eagle’s medium (DMEM, Life Technologies, Thermo Fisher Scientific, Loughborough, UK) and serum-free hormonally supplemented BEGM including 50 nM retinoic acid. Cultures were differentiated at air-liquid interface for at least 21 days with basal medium refreshment 5 times a week. The transepithelial resistance (TER) was measured in 14 fully differentiated ALI cultures using a voltmeter and compared between subjects below and above 45 years of age.

**Expression of *CDH1, EPCAM and TRPV4* in ALI cultures**

RNA was isolated from the control-treated ALI cultures used in Figure 1 (*EPCAM* and *TRPV4*) and Figure 3 (*CDH1*) of the paper “Inhibition of Pim1 kinase, new therapeutic approach in virus-induced asthma exacerbation” by *De Vries* et al[3]. ALI cultures were harvested in TRIzol reagent (Invitrogen, Paisly, UK) and total RNA was isolated following standard protocols. Contaminated DNA was enzymatically removed (DNA-free kit, Ambion, Austin, TX, USA). Reverse transcription was performed with iScript cDNA synthesis kit (Bio-Rad, Veenendaal, The Netherlands) for *CDH1* and with RevertAid First Strand cDNA Synthesis Kit (ThermoFisher Scientific) for *EPCAM* and *TRPV4*. For *CDH1* (Hs01023894_m1, ThermoFisher Scientific, Waltham, USA), mRNA expression levels were determined by RT-qPCR using 10 ng cDNA template and calculated relative to the average of the housekeeping genes *B2M* (Hs99999907_m1, ThermoFisher Scientific) and *PPIA* (Hs99999904_m1, ThermoFisher Scientific). For *EPCAM* (hs00901885_m1, ThermoFisher Scientific) and *TRPV4* (hs00540967_m1, ThermoFisher Scientific), mRNA expression levels were calculated relative to the housekeeping gene *B2M.*

**CRISPR-Cas9 knockout of *CDH1* in 16HBE14o- cells**

To evaluate the impact of loss of *CDH1* on barrier function, we used a heterozygous (CDH1^+/-^) and homozygous (CDH1^-/-^) CDH1 CRISPR-Cas9 knockout of the human bronchial epithelial cell line 16HBE 14o-. The CRISPR-Cas9 plasmids were designed using the online tool: <https://www.benchling.com/>. A 20-base long guide RNA (gRNA) was designed for both exon 1 (gRNA: 5’-GAGCCGCGACGACGACGACG-3’) and exon 2 (gRNA: 5’-CGATGTGCAAGTGCCACGGG-3’) targets from the *CDH1* gene sequence. The assembly of the plasmids was done by inserting the specific gRNA in expression vector PX245 (WT-2A-EGFP, 48138) containing ampicillin-resistant and green fluorescence protein (GFP) genes. Heat shock transformation was used to get the plasmids into *E.coli*, for amplification. The plasmid-positive *E.coli* were selected by their ampicillin resistance. The plasmids were isolated from *E.coli* and purified using SpinPlasmid Midi Kit (Qiagen) according to the manufacturer’s specification. 70-90% confluent 16HBE cell lines at passage 2 were transfected with the purified CRISPR-Cas9 plasmids using the Lipofectamine 3000 reagent (Invitrogen life technologies), according to the manufacturer’s specifications. The control cell line was transfected with plasmids without the gRNA. After an incubation period of 48 hours, the cells were harvested and FACS-sorted (with MoFlo© Astrios) to select the GFP-positive cells. Each GFP-positive cell was individually expanded by sub-culturing. The CRISPR-CAS9 knockout was validated by T7 PCR and Sanger sequencing of the DNA extracted from transfected 16HBE cells. Western blotting was used to confirm the knockout of *CDH1* (Supplementary Figure S2)

**16HBE14o- cell culture, ECIS and western blotting**
The human bronchial epithelial cell line 16HBE14o- was cultured in Eagle minimum essential medium (EMEM)/10% fetal calf serum (FCS; Bio Whittaker, Verviers, Belgium) on collagen-coated T25 flasks as described previously[4]. All culture media were supplemented with 100 U/ml penicillin and 100 mg/ml streptomycin. Cells were used for experiment between passages 70 and 75.

For ECIS, cells were seeded in 8 wells collagen-coated arrays at a density of 75000 cells in 400ul medium per well. The resistance and capacitance of cells were measured at 400Hz and 3200Hz respectively, using an ECIS machine (Applied Biophysics, Troy, NY, USA), for 48 hours.

For western blot, cells were seeded in a collagen-coated 24 wells plate at a density of 50000 cells in 1mL per well. They were grown to confluence and serum-deprived for 24 hours. Total cell lysates were obtained as previously described[4, 5]. Polyclonal Rabbit anti-human E-cadherin (1:1000; Santa Cruz Biotechnology, Heidelberg, Germany) and anti−human β-actin (1:1000; Santa Cruz Biotechnology, Heidelberg, Germany) were the primary antibodies used. Polyclonal goat anti-rabbit and rabbit anti-mouse immunoglobulins/HRP (1:2000; Dako Denmark, Flostrup, Denmark) was used as the secondary antibody. The blots were developed using SuperSignal^TM^ West Pico PLUS Chemiluminescent Substrate (cat#34577) according to the manufacturer’s guidelines (Thermo Fisher Scientific) and visualized using ChemiDoc XRS Imaging System running with Image Lab software (Bio-Rad Laboratories, California, United States).

**REFERENCES**

1. Boudewijn IM, Postma DS, Telenga ED, Ten Hacken NHT, Timens W, Oudkerk M, Ross BD, Galbán CJ, van den Berge M. Effects of ageing and smoking on pulmonary computed tomography scans using parametric response mapping. *Eur. Respir. J.* 2015; 46: 1193–1196.

2. Steiling K, van den Berge M, Hijazi K, Florido R, Campbell J, Liu G, Xiao J, Zhang X, Duclos G, Drizik E, Si H, Perdomo C, Dumont C, Coxson HO, Alekseyev YO, Sin D, Pare P, Hogg JC, McWilliams A, Hiemstra PS, Sterk PJ, Timens W, Chang JT, Sebastiani P, O’Connor GT, Bild AH, Postma DS, Lam S, Spira A, Lenburg ME. A dynamic bronchial airway gene expression signature of chronic obstructive pulmonary disease and lung function impairment. *Am. J. Respir. Crit. Care Med.* 2013; 187: 933–942.

3. Vries M de, Bedke N, Smithers NP, Loxham M, Howarth PH, Nawijn MC, Davies DE. Inhibition of Pim1 kinase, new therapeutic approach in virus-induced asthma exacerbations. *Eur. Respir. J.* 2016; 47: 783–791.

4. Heijink IH, Kies PM, Kauffman HF, Postma DS, van Oosterhout AJM, Vellenga E. Down-Regulation of E-Cadherin in Human Bronchial Epithelial Cells Leads to Epidermal Growth Factor Receptor-Dependent Th2 Cell-Promoting Activity. *J. Immunol.* 2007; 178: 7678–7685.

5. Chen Q, de Vries M, Nwozor KO, Noordhoek JA, Brandsma C-A, Boezen HM, Heijink IH. A Protective Role of FAM13A in Human Airway Epithelial Cells Upon Exposure to Cigarette Smoke Extract. *Front. Physiol.* 2021; 12: 690936.
