# Supplementary figures and images for: The relation between age and airway epithelial barrier function
Source: Respir Res. 2022 Mar 3;23:43. doi: 10.1186/s12931-022-01961-7 (PMC8892715; doi:10.1186/s12931-022-01961-7)

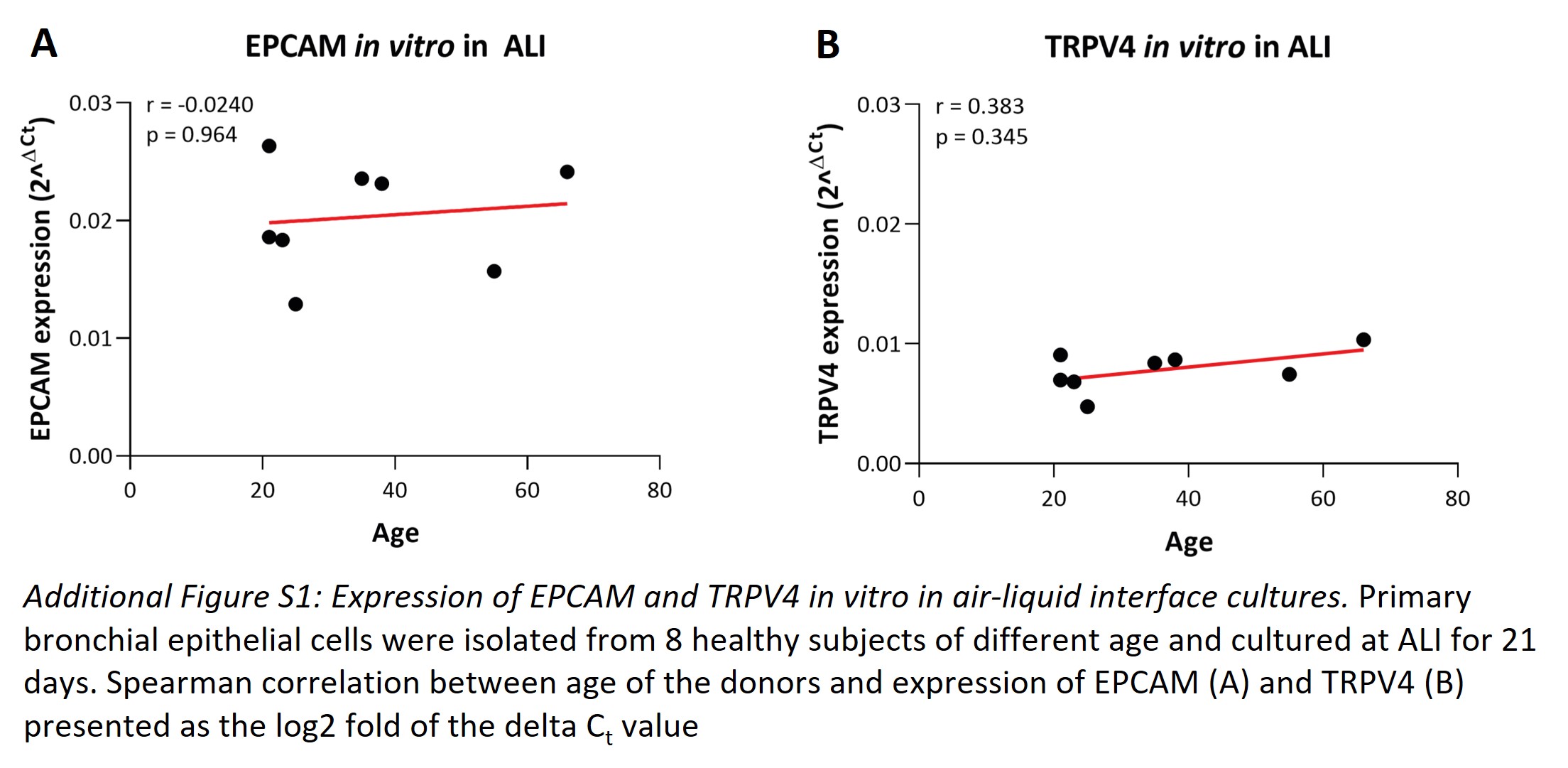

Supplement: Supplementary file 4 — Additional file 4: Figure S1. Expression of EPCAM and TRPV4 in vitro in air-liquid interface cultures. [file 12931_2022_1961_MOESM4_ESM.jpg]

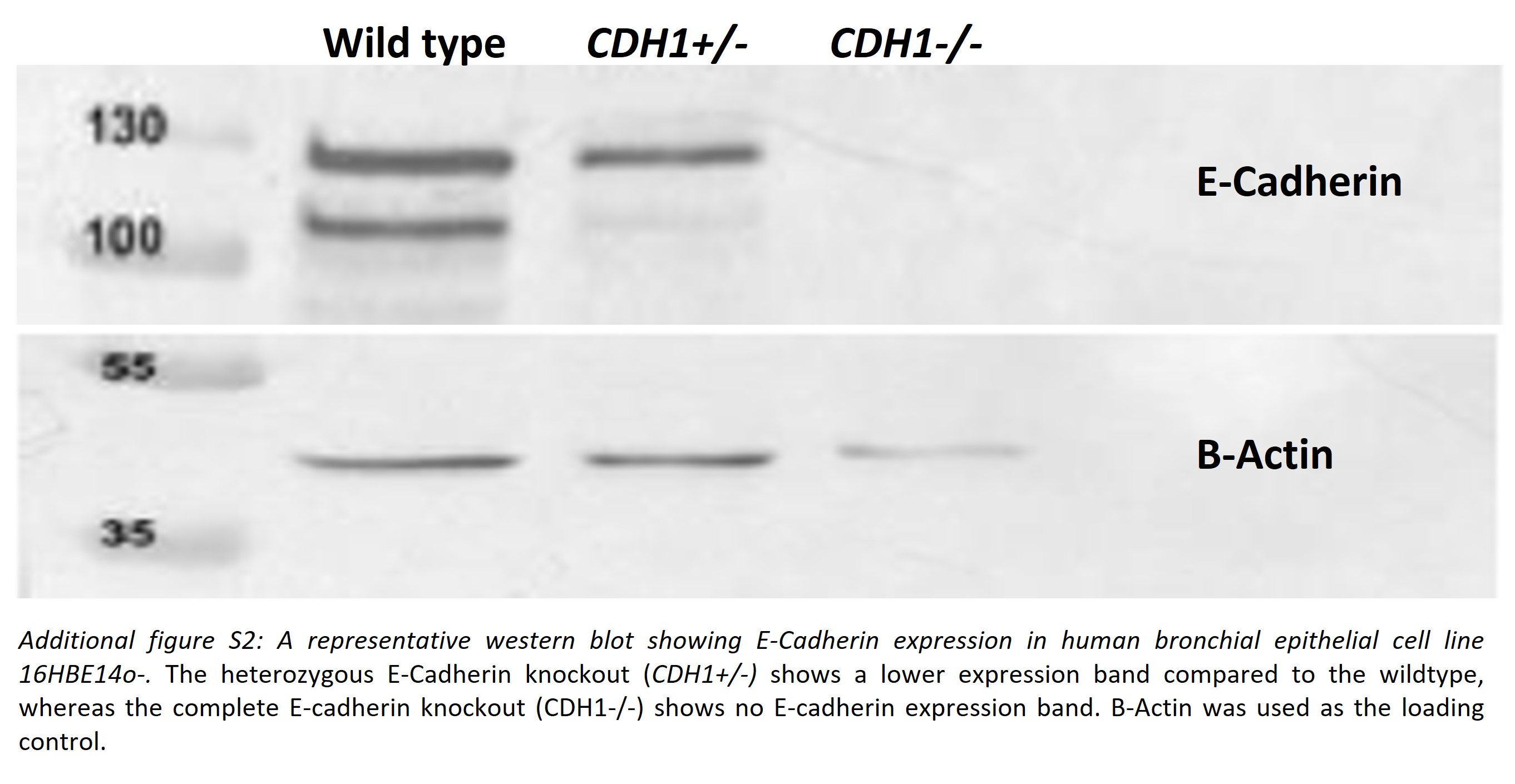

Supplement: Supplementary file 5 — Additional file 5: Figure S2. A representative western blot showing E-cadherin expression in human bronchial epithelial cell line 16HBE14o. [file 12931_2022_1961_MOESM5_ESM.jpg]

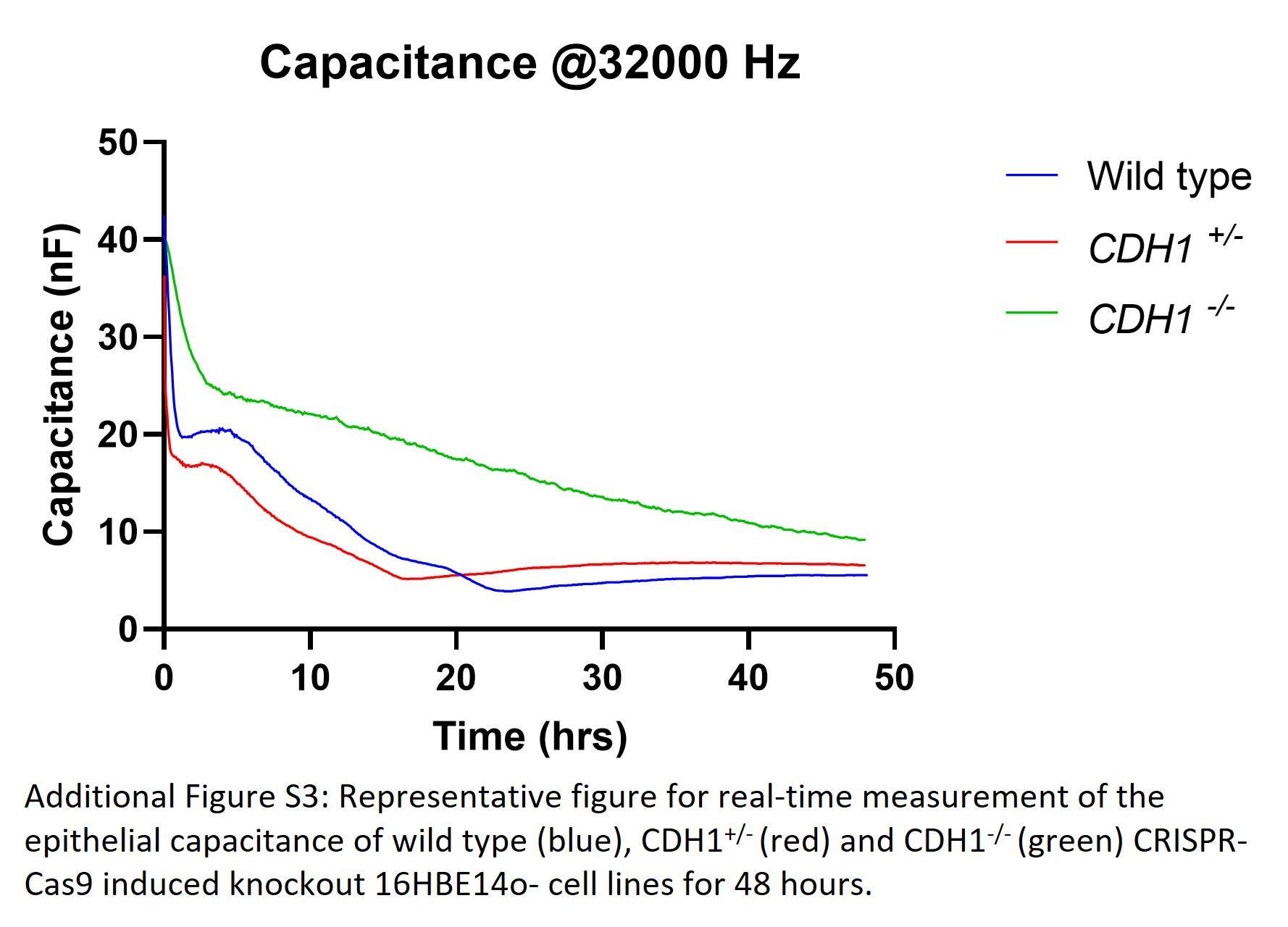

Supplement: Supplementary file 6 — Additional file 6: Figure S3. Representative figure for real-time measurement of the epithelial capacitance of wild type (blue), CDH1+/− (red) and CDH1−/− (green) CRISPR–Cas9 induced knockout 16HBE14o- cell lines for 48 h. [file 12931_2022_1961_MOESM6_ESM.jpg]
